# Prevalence and Sources of Disability-Based Discrimination in a National Sample of Graduating Medical Students

**DOI:** 10.1001/jamainternmed.2025.3148

**Published:** 2025-07-28

**Authors:** Mytien Nguyen, Justin L. Bullock, Karina Pereira-Lima, Tonya L. Fancher, Shruthi Venkataraman, Jung G. Kim, Srijan Sen, Jason Heath, Dowin Boatright, Lisa M. Meeks

**Affiliations:** 1 Department of Immunobiology, Yale University School of Medicine, New Haven, Connecticut; 2 Division of Nephrology, Department of Medicine, University of Washington School of Medicine, Seattle; 3 Department of Anesthesiology, University of Michigan Medical School, Ann Arbor; 4 Division of General Internal Medicine, University of California, Davis, School of Medicine, Sacramento; 5 Department of Emergency Medicine, New York University Langone Health, New York; 6 Institute for Innovations in Medical Education, New York University Langone Health, New York; 7 Department of Psychiatry, University of Michigan Medical School, Ann Arbor; 8 Department of Medical Education, University of Illinois College of Medicine, Chicago; 9 Department of Family Medicine, University of Michigan Medical School, Ann Arbor

## Abstract

This cross-sectional study describes experiences of discrimination among US medical students with disabilities as reported in a 2024 survey.

Experiences of discrimination during medical training are associated with burnout and attrition.^1,2^ While discrimination based on disability among physicians has been well-characterized,^3^ the prevalence and sources of disability-based discrimination remain unexplored among trainees, particularly across disability types, such as learning and psychological disabilities. With more individuals with disabilities entering medicine, understanding the prevalence and sources of disability-based discrimination is essential for promoting equity and retention in the profession.

## Methods

We used deidentified data from the 2024 Association of American Medical Colleges Graduation Questionnaire (GQ), which 80% of 2024 graduates completed. Self-reported disability status was categorized as nondisabled, learning, psychological, motor or sensory, chronic illness, multiple types, and other. The GQ asks all students the sources and frequency of experienced discrimination based on being denied opportunities for training or rewards based on a disability, receiving lower evaluations or grades solely because of a disability rather than performance, and being subjected to offensive remarks or names related to a disability (eAppendix in Supplement 1). We coded multiple disability–based discrimination as 2 or more discrimination experiences (eg, denied opportunities and subjected to offensive remarks). This cross-sectional study met the criteria from The New York University Langone Health Institutional Review Board for exemption with a waiver of informed consent because deidentified data were used. We followed the STROBE reporting guideline.

Descriptive statistics summarized discrimination experienced by disability type using Pearson χ^2^. Analyses were performed using Stata 18.0 (StataCorp). Two-sided *P* < .05 indicated statistical significance.

## Results

Among 1800 students with disability, 224 (12.4%) reported any disability-based discrimination. Students with chronic illness, motor or sensory disability, and multiple disabilities had the highest prevalence of being denied opportunities (26 [10.0%], 10 [8.1%], and 15 [13.8%], respectively), being subjected to offensive remarks (40 [15.3%], 16 [13.1%], and 24 [22.2%], respectively), and receiving lower evaluations (31 [11.9%], 12 [9.8%], and 16 [14.8%], respectively; all *P* < .001) (Table 1).

**Table 1.** Association of Disability Status With Reports of Disability-Based Discrimination

Disability status	Reports of discrimination, No. (%)										
	Total, No.	Denied opportunities for training or rewards based on a disability	*P* value	Subjected to offensive remarks/names related to a disability	*P* value	Received lower evaluations or grades based on a disability rather than performance	*P* value	Any disability-based discrimination	*P* value	Multiple disability–based discrimination	*P* value
All students with disability	1800	90 (5.0)	NA	157 (8.7)	NA	115 (6.3)	NA	224 (12.4)	NA	99 (5.5)	NA
Disability type^a^											
Learning	1108	27 (2.4)	<.001	55 (4.9)	<.001	42 (3.7)	<.001	78 (7.0)	<.001	33 (2.9)	<.001
Psychological	117	6 (5.1)		12 (10.2)		9 (7.6)		18 (15.3)		7 (5.9)	
Motor or sensory	122	10 (8.1)		16 (13.1)		12 (9.8)		22 (18.0)		10 (8.1)	
Chronic illness	260	26 (10.0)		40 (15.3)		31 (11.9)		62 (23.8)		27 (10.3)	
Other	85	6 (7.0)		10 (11.7)		5 (5.8)		13 (15.2)		6 (7.0)	
Multiple type	108	15 (13.8)		24 (22.2)		16 (14.8)		31 (28.7)		16 (14.8)	

Abbreviation: NA, not applicable.

^a^ Learning disability includes all learning disabilities and attention-deficit/hyperactivity disorder. Motor or sensory disabilities include vision, hearing, and mobility disability.

Clinical clerkship faculty, residents, and administrators were the most commonly cited sources of denied opportunities (53 [58.9%], 26 [28.9%], and 23 [25.6%], respectively). Clinical clerkship faculty and residents were the most commonly cited sources of lower evaluations (86 [74.8%] and 42 [36.5%]). Clinical clerkship faculty, residents, and students were the most commonly cited sources of offensive remarks (81 [51.6%], 48 [30.6%], and 31 [19.8%], respectively) (Table 2).

**Table 2.** Sources of Disability-Based Discrimination

Source	Reports of discrimination, No. (%)		
	Denied opportunities for training or rewards based on a disability (n = 90)	Subjected to offensive remarks or names based on a disability (n = 157)	Received lower evaluations or grades based on a disability rather than performance (n = 115)
Students	7 (7.8)	31 (19.8)	3 (2.6)
Preclinical faculty	11 (12.2)	21 (13.4)	13 (11.3)
Clerkship faculty (didactics)	14 (15.6)	13 (8.3)	15 (13.0)
Clerkship faculty (clinical)	53 (58.9)	81 (51.6)	86 (74.8)
Residents	26 (28.9)	48 (30.6)	42 (36.5)
Nurses	6 (6.7)	17 (10.8)	3 (2.6)
Administrators	23 (25.6)	25 (15.9)	14 (12.2)
Other institutional employees	14 (15.6)	20 (12.7)	8 (7.0)

## Discussion

To our knowledge, this is the first study to describe the prevalence of disability-based discrimination in a large, national sample of medical students. Students with chronic illness, motor or sensory disability, and multiple disabilities reported the highest discrimination rates. This finding aligns with prior studies demonstrating that fear of stigma and bias often discourages trainees from disclosing disabilities or seeking accommodations, perpetuating a cycle of silent struggle that limits access to essential resources for success.^4^ While offensive remarks related to disability were reported less frequently than those based on gender, race, and sexual orientation in prior work,^5^ prevalence was similar for denied opportunities and lower evaluations.

Clinical faculty and residents were the most frequent sources of discrimination reported. This finding aligns with prior research demonstrating that clinical faculty and residents are primary sources of mistreatment during medical school,^6^ which may explain the high rates of denied opportunities and lower evaluations from these sources. These challenges are further compounded by difficulties students encounter in requesting reasonable accommodations, including unclear implementation guidelines, inconsistent enforcement, and lack of institutional support.^4^ To address these issues, programs should foster an inclusive clinical educational culture, where students are empowered to report discrimination without fear of retaliation, train faculty and residents on disability awareness and inclusive teaching strategies, and learning happens in universally designed settings.

Study limitations include self-reported and cross-sectional data, which may underestimate prevalence of discrimination and introduce uncertainty about whether discriminatory experiences stem from disability or other factors. However, students with disabilities are often attuned to how their disability shapes their experiences and can recognize subtle bias patterns.^4^ Future studies should investigate the long-term implications of disability-based discrimination for educational and professional outcomes.

## Notes

Supplement 1. **eAppendix.** Survey Questions

Supplement 2.
Data Sharing Statement
