# Supplementary material for: Prevalence and Sources of Disability-Based Discrimination in a National Sample of Graduating Medical Students
Source: JAMA Intern Med. 2025 Jul 28;185(9):1164–6. doi: 10.1001/jamainternmed.2025.3148 (PMC12305438; doi:10.1001/jamainternmed.2025.3148)
Supplement: Supplement 1. — eAppendix. Survey Questions [file jamainternmed-e253148-s001.pdf]

Supplemental Online Content

Nguyen M, Bullock JL, Pereira-Lima K, et al. Prevalence and sources of disability-based discrimination in a national sample of graduating medical students. *JAMA Intern Med.* Published online July 28, 2025. doi:10.1001/jamainternmed.2025.3148

eAppendix. Survey Questions

This supplementary material has been provided by the authors to give readers additional information about their work.

© 2025 American Medical Association. All rights reserved, including those for text and data mining, AI training, and similar technologies.

eAppendix. **<u>Survey questions</u>**

Questions on disability and disability discrimination on the AAMC Graduation Questionnaire

Source: https://www.aamc.org/data-reports/students-residents/report/graduation-questionnaire-gq

Are you a person with a disability (e.g., ADHD, learning, psychological, chronic health, mobility, hearing, vision, etc.)?

- Yes
- No
- I do not know

Which of the following describes your disability? If you have more than one type, select all that apply.

- Attention deficit/hyperactivity disorder
- Chronic health disability (please specify):
- Deaf or hard of hearing
- Learning disability (please specify):
- Mobility disability
- Psychological disability (please specify):
- Visual disability
- Another disability or condition (please specify):

For each of the following behaviors, please indicate the frequency you personally experienced that behavior during medical school. Include in your response any behaviors performed by faculty, nurses, residents/interns, other institution employees or staff, and other students. Please do not include behaviors performed by patients.
During medical school, how frequently have you...(Options: Never, Once, Occasionally, Frequently)

- Been denied opportunities for training or rewards based on a disability?
- Been subjected to offensive remarks/names related to a disability?
- Received lower evaluations or grades solely because of a disability rather than performance?

You indicated that you personally experienced the following behavior(s) during medical school:

Indicate below which person(s) engaged in the behavior that was directed at you. Check all that apply.

© 2025 American Medical Association. All rights reserved, including those for text and data mining, AI training, and similar technologies.

- Preclerkship faculty
- Clerkship faculty (classroom)
- Clerkship faculty (clinical setting)
- Resident/Intern
- Nurse
- Administrator
- Other institution employee
- Student

© 2025 American Medical Association. All rights reserved, including those for text and data mining, AI training, and similar technologies.
