# Supplementary material for: Prevalence and Sources of Disability-Based Discrimination in a National Sample of Graduating Medical Students
Source: JAMA Intern Med. 2025 Jul 28;185(9):1164–6. doi: 10.1001/jamainternmed.2025.3148 (PMC12305438; doi:10.1001/jamainternmed.2025.3148)
Supplement: Supplement 2. — Data Sharing Statement [file jamainternmed-e253148-s002.pdf]

Data Sharing Statement

Nguyen. Prevalence and Sources of Disability-Based Discrimination in a National Sample of Graduating Medical Students. *JAMA Intern Med*. Published July 28, 2025. doi:10.1001/jamainternmed.2025.3148

Data
Data available: No
